# Does the Association between Depressive Symptomatology and Physical Activity Depend on Body Image Perception? A Survey of Students from Seven Universities in the UK

**DOI:** 10.3390/ijerph8020281

**Published:** 2011-01-25

**Authors:** Walid El Ansari, Christiane Stock, Ceri Phillips, Andi Mabhala, Mary Stoate, Hamed Adetunji, Pat Deeny, Jill John, Shan Davies, Sian Parke, Xiaoling Hu, Sherrill Snelgrove

**Affiliations:** 1 Faculty of Applied Sciences, University of Gloucestershire, Gloucester GL2 9HW, UK; 2 Unit for Health Promotion Research, Institute of Public Health, University of Southern Denmark, 6700 Esbjerg, Denmark; E-Mail: cstock@health.sdu.dk; 3 School of Human and Health Sciences, Swansea University, Swansea SA2 8PP, Wales, UK; E-Mails: c.j.phillips@swansea.ac.uk (C.P.); shan.davies@swansea.ac.uk (S.D.); j.e.john@swansea.ac.uk (J.J); s.parke@swansea.ac.uk (S.P.); s.r.snelgrove@swansea.ac.uk (S.S.); 4 Faculty of Health and Social Care, University of Chester, Chester CH1 4BJ, UK; E-Mail: a.mabhala@chester.ac.uk; 5 School of Science, Society and Management, Bath Spa University, Bath BA2 9BN, UK; E-Mail: m.stoate@bathspa.ac.uk; 6 School of Health & Social Care, Oxford Brookes University, Oxford OX3 0FL, UK; E-Mail: hadetunji@brookes.ac.uk; 7 Institute of Nursing Research, School of Nursing, University of Ulster, Londonderry, Northern Ireland BT48 7Jl, UK; E-Mail: pg.deeny@ulster.ac.uk; 8 Business School, University of Gloucestershire, Cheltenham GL50 2RH, UK; E-Mail: xhu@glos.ac.uk

**Keywords:** depression, physical activity, student health, university, college, gender, body image

## Abstract

This cross-sectional study assessed the association between depression and PA in university students of both genders and the role of body image perception as a potential effect modifier. Undergraduate students (N = 3706) from seven universities in the UK completed a self-administered questionnaire that assessed sociodemographic information; a range of health, health behaviour and health awareness related factors; the modified version of Beck’s Depression Inventory (M-BDI); educational achievement, and different levels of physical activity (PA), such as *moderate* PA (at least 5 days per week moderate exercise of at least 30 minutes), and *vigorous* PA (at least 3 days per week vigorous exercise of at least 20 minutes). Only 12.4% of the sample achieved the international recommended level for moderate PA, and 33.1% achieved the recommendations for vigorous PA. Both moderate and vigorous PA were inversely related to the M-BDI score. Physically active students, regardless of the type of PA, were significantly more likely to perceive their health as good, to have higher health awareness, to perform strengthening exercises, and to be males. The stratified analyses indicated that the association between depression and PA differed by body image. In students perceiving their body image as ‘just right’, moderate (>4th percentile) and high (>5th percentile) M-BDI scores were inversely related to vigorous PA. However, in students who perceived their body image as ‘overweight’, the inverse association was only significant in those with high M-BDI scores. We conclude that the positive effect of PA on depression could be down modulated by the negative impact of a ‘distorted’ body image on depression. The practical implications of these findings are that PA programmes targeting persons with depressive symptoms should include effective components to enhance body image perception.

## 1. Introduction

Physical activity (PA) seems related to mental health and wellbeing. Since some decades, exercise has been suggested to be associated with better mental health [1,2]. Similarly, wellness has been described as a concept that underlines the importance of PA for the feeling of wellbeing [3].

Unsurprisingly, in recent years, studies have explored the role of PA as a potential component in the prevention and/or management of depressive symptoms [4]. Reviews of observational and intervention studies examined the links between PA and depression/depressive symptoms [5–7]. Several appraisals indicated parallel conclusions: that PA was positively associated with reduced likelihood of depression or depressive symptoms [8]. Indeed, observational [9–11] and intervention research [12,13] found that higher levels of PA (e.g., >60 minutes of PA per week) were associated with lower odds of depression. Moreover, others [14–16] concluded that even low levels of PA (e.g., exercising as little as 30 minutes per week) were associated with enhanced mental health.

Nevertheless, cross-sectional [17–19] and prospective inquiries [20–22] suggested conflicting inconsistencies on the association between PA and depression. On the one hand, three community-based longitudinal inquiries concluded that PA was not protective for subsequent depression [23–25]. However the samples examined in these studies were not homogenous: Cooper-Patrick *et al.* [23] and Lennox *et al.* [24] examined only middle-aged men, whilst Weyerer [25] included males and females as well as older subjects. On the other hand, another three community-based inquiries [20,21,26] found a protective effect of PA on depression. Again, the samples were not homogenous: Paffenbarger *et al.* [26] limited their inquiry to male college students; Camacho *et al.* [21] and Farmer *et al.* [20] included men and women of a very wide age range, and whilst the former reported a protective effect of PA on depression for both men and women, the latter found an effect only for females.

The inconsistent findings of these studies draw attention to the need for a range of confounding features related to depression (e.g., unhealthy lifestyle), along with other aspects to be taken into account when assessing the links between physical exercise and well-being [10,27]. Thus, in gauging the association between PA and depressive symptoms, several demographic, behavioural, social, and academic factors need to be simultaneously considered [28].

*Gender:* research [26] has suggested that moderate to high levels of weekly energy expenditure in men were associated with decreased likelihood of depression. Conversely, others [20] reported a protective effect of PA on depression only for women. Thus, the current study included male and female university students as participants.

*Self-rated health status (perceived health)*: in the U.S.A., high-school students’ self-reported health status was modestly correlated (*r* 0.09 to 0.22) with five life domains (satisfaction with family, friends, school, living environment, and self) and overall life satisfaction (r = 0.21) [29]. In relation to one another, depressive symptoms were more strongly associated with self-reported mental health status, while PA was more strongly linked with self-reported physical health [30]. Perceived health incorporates physical, emotional and personal components of health that collectively make up individual “healthiness” ([14], p. 242). As such, perceived health seems a broader indicator of health-related wellbeing. Some authors [10] have proposed a relationship between PA and decreasing depressive symptoms in middle-aged women, independent of pre-existing physical and psychological health. Hence we included self-rated health status in the current investigation of the links between PA and depressive symptoms.

*Health awareness*: from the mental health perspective, teens in primary care settings were not seeking mental health care even when depression was detected, suggesting that such adolescents may be at different stages of recognition of their conditions [31], or might benefit from more awareness. Likewise, many persons with schizophrenia were unaware of the symptoms and consequences of their condition, and such unawareness was a risk factor for poor adherence to treatment and poor outcomes [32]. From the PA perspective, there is a need for a greater understanding of the (perhaps yet anticipated) antecedents of PA participation [33]. In such context, enhanced health awareness/consciousness could play a role as determinants of an active lifestyle and in negotiating a physical identity, and could be drivers for a commitment towards PA. Indeed, a proposed explanation for the limited effectiveness of PA interventions is that people may lack awareness of their health behaviour [34]. As health awareness appeared to be related to mental health and PA, we included it in the current investigation of the PA-depressive symptoms relationships.

*Educational achievement*: education or academic achievement seem interrelated with both PA and mental health. In relation to PA, a link between physical fitness and academic achievement in elementary school children has been suggested [35]. In Swedish 9th-grade pupils, academic achievement was associated with vigorous PA in girls [36]. In the USA, promoting fitness of school pupils by increasing their PA opportunities enhanced the pupils’ academic achievement even after controlling for potentially confounding factors [37].

As regards mental health, exercise could be a simple yet significant way of enhancing those aspects of children’s mental functioning central to cognitive development [38]. The well educated tend to be mentally and physically healthier than the less well educated [39,40], and, poor academic achievement is a risk for later depression [41]. The association between depressive symptoms during adolescence and educational attainment in young adulthood [42] indicate that depressive symptoms were associated with higher odds of failure to complete high school (only for girls). Among high school graduates (both genders), depressive symptomatology was associated with failure to enter college.

Furthermore, in connection with body image, cognitive development in adolescents could be linked to weight perception and body image [43]; and tools that measure depressive symptoms (e.g., Modified Beck Depression Inventory, MBDI) include questions about one’s appearance [44–46]. Despite this, surprisingly, El Ansari and Stock [47] recently examined a sample of UK university students and found that neither PA nor depressive symptoms were significantly associated with three different subjective and objective measures of academic achievement. Notwithstanding, there have been calls to examine the relationship between mental health problems and factors such as educational attainment, that impact physical health later in life [42]. We included educational achievement in assessing the relationships between PA and depressive symptoms.

*Strengthening or toning exercises*: PA appears to be inversely related to the risk of depression [8]. However, although leisure-time PA is inversely associated with depression among females [48,49], few have explored the association with PA undertaken in other domains (e.g., work-, domestic- and transport-related) [50]. Others failed to control for involvement in non-aerobic activity [51–53], or other forms of PA e.g., strengthening or toning exercises. This is despite that among healthy older adults, resistance training has been associated with improved mood states [54]; and moderate- or high-intensity muscle-strengthening activities two or more days a week provided additional health benefits [55]. Interpretive difficulties arise due to such lack of control for the potential factors that could influence the PA-depression relationship. This suggested that it was important to include strengthening or toning exercises in the current study of the links between PA and depressive symptoms.

*Intensity of physical activity* (*dose-response relationships between PA and depressive symptoms*): Several studies reported lower depressive symptomatology among physically active males and females [56–58]. In support, longitudinal, cross-sectional and intervention research [15,18,54] found significant inverse associations between moderate-intensity PA and odds of depression. Fewer studies examined the relative strengths of associations between moderate or high intensity PA and the risk of depression [59,60]. A follow up of 21,596 men for 20 years showed a dose-response relationship between PA and physician-diagnosed depression [26]. Recently, links between increasing PA and declining depressive symptoms in middle-aged females, independent of pre-existing physical/psychological health have been suggested [10]. Hence in the present study, we included several forms (levels) of PA: *low; moderate*; and *vigorous*.

*Body image*: the role of PA in a health-seeking life style seems increasingly about the improvement of one’s silhouette, where for instance, many women exercise in order to achieve a better silhouette [61]. Most cultures assign importance to appearance [62]. In Western traditions, being slim and fit signifies a high marketing value of self-control and personal success [63]. Moreover, obesity appears associated with depression, low self-esteem, and poorer quality of life [64,65]. Negative social and emotional associations of overweight and obesity e.g., low self-esteem, depression, behavioural and learning problems have also been suggested [66–68]. There is some evidence of greater risk of depression among the obese [69–71].

In line with others [5], for this paper, we use the terms ‘depression/depressive symptoms/depressive symptomatology’ variously to describe a dysphoric mood state, a syndrome of a cluster of symptoms (e.g., sadness, fatigue, loss of appetite, disturbed sleep, disappointment with oneself and other self-reported experiences). PA was defined as “any bodily movement produced by skeletal muscles that result in energy expenditure” ([72], p.126).

*Aim of the Study:* Based on the above background, the main aim of the current study was to assess the association between depression and PA in university students of both genders. We assumed that PA is inversely associated with depression even when controlled for other factors. In order to control for a range of factors that have previously been shown to be associated with PA as well as with depression (gender; perceived health; educational achievement; strengthening or toning exercises; and body image perception), these variables were included into the analysis in order to control for potential confounders.

We examined three different levels of PA (low, moderate and vigorous) as dependent variables in order to analyse any dose-response differences in the association based on level of PA. We hypothesized that university students who achieved recommended guidelines of vigorous PA would exhibit lower levels of depressive symptomatology than students who achieved recommended levels of moderate PA. The highest level of depression would be expected in students not engaged in any level of PA.

A second sub aim of the analysis was to study the role of body image perception as a potential effect modifier of the relationship between PA and depressive symptomatology. We hypothesized that the inverse association between depression and PA might be stronger in students who perceived their body image as ‘just right’ when compared to students who perceived their body image as ‘overweight’.

## 2. Methods

### 2.1. Sample and Data Collection Procedures

The study received ethical approval by research and ethics committees at the participating institutions. A representative sample of students was sought at all participating universities by selecting courses adequately representing the different departments/faculties, where self-administered questionnaires were distributed to all participants attending the selected classes/courses, and then collected after completion. Students were informed that participation was voluntary and anonymous, and that by completing the questionnaire, they agree to participate in the study. No incentives were provided for participation. Each questionnaire included a participant information sheet outlining the research aims and objectives. Students who remained in class to participate read the information sheet and, if they wished to participate, removed and kept it for future reference. In this manner it was ensured that the students who stayed in class to complete the survey consented to their participation and were not unwittingly coerced into the survey. Data were confidential and data protection was observed at all stages of the study. All data were computer-entered centrally at one site thus maximising the quality assurance while minimising potential data entry errors.

Data were collected during the academic year 2007–2008 at seven universities in England. The total sample comprised 3705 undergraduate students from England [University of Gloucestershire (n = 970, 43.6%_♂_, mean age 23.3 ± 8.4 years), Bath Spa University (n = 485, 22.6%_♂_, age 22.2 ± 6.9), Oxford Brookes University (n = 208, 10.8%_♂_, age 31.6 ± 10.4), University of Chester (n = 993, 13.1%_♂_, age 26.0 ± 9.2), Plymouth University (n = 169, 56.2%_♂_, age 24.6 ± 7.2)]; Wales [University of Swansea (n = 406, 7.8%_♂_, age 25.0 ± 7.4)]; and Northern Ireland [University of Ulster (n = 474, 8.2%_♂_, age 25.2 ± 7.7)]. Based on the number of returned questionnaires, the response rates were ≈ 80%.

### 2.2. Health and Wellbeing Questionnaire

The study was a general student health and wellbeing survey similar to studies of student health implemented in several countries [73,74] and comprised 133 items with an average 15–20 minutes completion time. It included socio-demographic information (e.g., gender, age), self-reported health data, as well as questions of health behaviours (nutrition, PA, smoking and alcohol consumption), social support, and university study related questions.

*Vigorous exercise* (1 item): students were asked: “On how many of the past 7 days did you participate in vigorous exercise for at least 20 minutes?” Participants answered with a rating from 0 to 7 days. A cut-off of at least 3 days a week was used in the analysis based on guidelines of the American Heart Association (AHA) for vigorous PA [75].

*Moderate exercise* (1 item): “On how many of the past 7 days did you participate in moderate exercise for at least 30 minutes?” Participants answered with a rating that ranged from 0 to 7 days. A cut-off of at least 5 days a week was used in the analysis based on AHA guidelines for moderate PA [75].

*Strengthening or toning muscles* (1 item): “On how many of the past 7 days did you do exercises to strengthen or tone your muscles, such as push-ups, sit-ups, or weight lifting?” Participants answered with a rating that ranged from 0 to 7 days. The cut-off of at least 2 days a week was used in the analysis based on AHA recommendations [75].

*Depressive symptoms*: were measured using the Modified Beck Depression Inventory (M-BDI) [44–46]. The modification of the original BDI included two approaches: (a) the four items per symptom which assessed the specific symptom’s intensity in the original BDI were replaced by a single statement per symptom with a six point Likert scale measuring its frequency in the last 4 weeks (past few days in our questionnaire) (with the two extreme categories labelled as 0 = ‘Never’, 5 = ‘Almost Always’). Sample items include: “I feel sad”, “I feel I am being punished”, “I have thoughts of killing myself”, “I have lost interest in other people”, “I have to force myself to do anything”, “I am worried about my appearance”, “I have no appetite”, and, “I lost interest in sex”. The reduction in the number of items per symptom is consistent with another recent modification of BDI (BDI-II) [46]. The versions of the BDI compute a single score for individual respondents by summing their responses for all items of the scale. Through a German sample reflecting the general population and in selected subsamples [44,45], the authors of the M-BDI demonstrated its construct validity and measurement equivalence as compared to the original BDI. They also provided a cut-off score for screening for clinically relevant depressive symptoms at ≥35, corresponding to the 85th percentile of the representative sample of the German population [76].

*Body image perception* (1 item): assessed on a five-point Likert scale adapted from the Health Behavior in School-aged Children (HBSC) study [77] and recently used by others, e.g., El Ansari *et al.* [74] after a change of the word “fat” of the item to “overweight”, as it was felt that the latter term is less judgmental. Students were asked: “In your opinion are you…”, with five response options (“Far too thin”, “A little too thin”, “Just right”, “A little overweight”, “Very overweight”). For the analysis, the five options were re-coded into three binary variables (“Underweight”, “Just right”, “Overweight”).

*Self-rated health status* (1 item): “How would you rate your health in general?” with a 5-point scale response format (1 = “excellent”, 2 = “very good”, 3 = “good”, 4 = “fair”, 5 = “poor”) as used in the German Federal Health Survey [78] (similar wording used by American College Health Association [79].

*Health awareness*: (1 item): as used by others [80], an item asked students about their general awareness of their health: “To what extent do you keep an eye on your health?”, with a four-point response scale (1= ‘not at all’ to 4= ‘very much’)

*Educational achievement* (1 item): ): assessed on a five-point Likert scale adapted from the Health Behavior in School-aged Children (HBSC) study [77]. As our sample comprised university students, we modified the original item (how teacher rated the pupil’s performance) to a self-rating: “How do you rate your performance in comparison with your fellow students?” (five response categories, from 1 = ‘much worse’ to 5 = ‘much better’) [47].

### 2.3. Statistical Analysis

We used SPSS 14.0 (SPSS Inc. Chicago, IL) to calculate frequencies and proportions and to conduct the statistical analyses. In order to present prevalences of students performing different levels of PA by university taking into account the varying male-to-female ratio of the samples at the different sites, we sex-adjusted the prevalences using direct standardization towards a male-to-female ratio of 30% to 70%. For several variables, some of the response options were combined to satisfy the assumption of adequate cell size for regression analysis. We performed multifactorial logistic regression analysis with enter mode in order to examine odds ratios for the association between depressive symptoms and different forms of PA (low, recommended moderate, recommended vigorous) as dependent variables after adjusting for potential confounding factors (gender, perceived health, health awareness, muscle strengthening, academic performance). While vigorous and moderate PA were defined as stated above, low PA was defined, in line with others, as “not participating in any vigorous or moderate PA or participating in only one day of moderate PA” [81]. We then stratified the analysis according to perceived body image (as ‘just right’ or as ‘overweight’) in order to explore whether perceived body image modified the association between depressive symptoms and different forms of PA. The ‘underweight’ category of perceived body image was not included in the stratified analysis due to inadequate sample size in this category. In order to determine the goodness-of-fit of each model to the survey data we used the Hosmer-Lemeshow test.

## 3. Results

### 3.1. Prevalence Levels of Recommended PA and of Associated Factors

Table 1 shows that only 12.4% of our sample of students achieved the American College of Sport Medicine and AHA’s recommended guidelines [75] of moderate PA per week, while 33.1% achieved the recommended levels of vigorous PA. Roughly 20.6% had a depression score >80th percentile. Every second student perceived their own health as ‘very good’ or ‘excellent’, and the majority (84%) had health awareness and reported that they ‘kept an eye’ on their health. A small proportion (15.8%) rated their own academic performance at university as better than that of their peers. Slightly more than half the participants (59.6%) perceived their body image as ‘overweight’.

### 3.2. Factors Associated with Physical Activity

Table 2 shows the results of multifactorial logistic regression models for the factors associated with three different levels of PA as the dependent variables. Academic performance was not associated with any type of PA. Both forms of PA at recommended level were inversely related with the M-BDI score >5th quintile, but vigorous PA was additionally inversely related with M-BDI score at moderate level (>4th quintile). Students achieving PA guidelines, regardless of the type of PA, were significantly more likely to perceive their health as good (better than those who did not achieve the PA guidelines), to have higher health awareness/consciousness, to perform strengthening exercises, and to be males. In contrast, students with low PA level were more likely to be older, to have lower levels of perceived health and health awareness, and were less likely to perform strengthening exercise. In contrast to students being physically active at recommended level, being low active was not associated with depressive symptoms or with gender.

### 3.3. Factors Associated with PA by Body Image Perception

Table 3 shows the results of multifactorial logistic regression models for factors associated with three different levels of PA as dependent variables by body image perception (as ‘just right’ or ‘overweight’). The stratified analyses indicated that the association between depression and PA differed by body image:

- In students perceiving themselves as just right, moderate (>4th percentile) and high (>5th percentile) M-BDI scores were inversely related with vigorous PA. However, in students who perceived themselves as overweight, the inverse association was only significant in those with high M-BDI scores.

Other factors associated with PA differed also by body image:

- Academic performance was positively associated with low PA in students perceiving themselves as just right and negatively in students perceiving themselves as overweight
- In students perceiving themselves as just right, male gender was associated only with vigorous PA. However, in students who perceived themselves as overweight, this association was significant for both forms of recommended PA (moderate and vigorous).
- In students perceiving themselves as just right, perceived health was only inversely associated with low PA. However, in students who perceived themselves as overweight, this association was significant for both forms of recommended PA but not for low PA.
- In students perceiving themselves as just right, health awareness was associated with any of the three forms of PA. However, in students who perceived themselves as overweight this association was only significant in low (inverse) and vigorous PA.

## 4. Discussion

We examined the triad of associations that are important for university students: depressive symptomatology, PA and body image perception. The current study investigated three different levels of PA (low, moderate and vigorous); two levels of body image perception (‘just right’ and ‘overweight’); and controlled for potential confounders shown to be associated with PA as well as with depression (age, gender, perceived health, health awareness, educational achievement, and strengthening or toning exercises). Given that investigating a few isolated variables obscures confounding relationships, this study links up these various features in order to capture the wider picture of the links between depressive symptoms and PA in university students. In understanding those links, it is important to control for a range of variables, as others have found that although the risk of depression can be altered by changes in exercise habits, these associations were not statistically significant after adjustment for covariates [19].

### 4.1. Prevalence Levels of Recommended PA and of Associated Factors

Roughly 12.4% and 33.1% of our sample of students achieved the recommended AHA guidelines [75] of moderate and vigorous PA levels of PA per week respectively. Both these values compared unfavourably with recent PA levels of college students from 4 universities in 3 Midwestern states in the USA, where 39% and 56% of the total respondents met the moderate and vigorous guidelines respectively (*i.e.*, 56% of the total respondents participated in at least 20 minutes of vigorous physical activity on 3 or more days of the past 7 days, and participated in at least 30 minutes of moderate physical activity 5 or more days of the past 7 days) [81]. This discrepancy might be related to the samples’ characteristics. Seo *et al.*’s [81] participants were from a group of students who were enrolled in health-related courses, suggesting that those students might have had a pre-existing interest in health behaviours or that their current engagement in the course had influenced their activity levels. Although our sample had the majority of students from health related courses (e.g., nursing or health and sport related, there were other participants from other courses e.g., psychology and business courses). However, the levels recorded by our sample also seemed lower than the national estimates reported by researchers who used the 1995 National College Health Risk Behavior Survey, where 20% met the moderate guideline and 38% for met the vigorous guideline [82].

As regards strengthening exercises (≥twice per week), whilst 23.9% of our sample reported such exercises at least twice per week, such levels compared unfavourably with the 47.7% of students in the USA who reported that they exercised to strengthen or tone muscles at least 2 out of the past 7 days [83]. In connection with depressive symptomatology, roughly 20.6% of our sample exhibited a depression score >80th percentile. These levels compared favourably with university students in a range of western and eastern European countries (Poland, Bulgaria, Denmark and Germany) [84]. The proportion of students from those countries with MBDI scores ≥35 (corresponding to the 85th percentile) was above 40% (female students in Poland and Bulgaria), to around 25% (female students in Denmark and Germany); and for male students it fluctuated (22.8%-Germany, 12.1%-Denmark, 27.3%-Poland and 33.8%-Bulgaria) [84].

### 4.2. Factors Associated With Physical Activity

In connection to our research aim to assess the association between depression and PA, we expected to find a significant inverse association even when controlling for other factors. The results of our analysis showed that students performing PA at recommended level were less likely to have an M-BDI score >80th percentile, a limit that is an accepted indicator for depression, independent of potentially confounding factors. Therefore our findings support a protective effect of PA, or that physical inactivity may be a risk factor for depressive symptoms, which is in line with other cross-sectional [17–19] and prospective studies [20–22]. Although some longitudinal studies did not clearly demonstrate protective effects of PA programmes (see introduction) this can be result of ineffective programmes or weak implementation and does not necessarily contradict our findings. It is important to note however, that from our cross-sectional study we cannot conclude any cause-effect relationship: PA may lower depression or depression may lower PA.

Regarding the research aim to study dose-response relationship between PA and depression, we hypothesized that university students who achieve recommended guidelines of vigorous PA would exhibit lower levels of depressive symptomatology than students who achieve recommended levels of moderate PA. The highest level of depression would be expected in students not engaged in any level of PA. Our findings are supporting these hypotheses. While low PA level was not associated with depression, the protective effect of PA increased with PA intensity. Both forms of PA (moderate and vigorous) were inversely related with the M-BDI score above the 5th quintile, but vigorous PA was additionally inversely related with M-BDI score at moderate level (>4th quintile). These results suggested that the association between PA and depression may increase with PA intensity, which has also been found in some few other studies [26,59,60].

### 4.3. Factors Associated with Physical Activity by Body Image Perception: Effect Modification?

A sub aim of the analysis was to study the role of body image perception as a potential effect modifier of the relationship between PA and depressive symptomatology. We hypothesized that the inverse association between depression and PA might be stronger in students who perceive their body image as ‘just right’ when compared to students who perceive their body image as ‘overweight’. In line with this hypothesis we found that in students perceiving themselves as just right, moderate (>4th quintile) and high (>5th quintile) M-BDI scores were inversely related with vigorous PA. However, in students who perceived themselves as overweight, the inverse association was only significant in those with high M-BDI scores. This might be explained in the way that a ‘distorted’ body image would down modulate the inverse association between depression and PA. In other words, the positive effect of PA on depression would be down modulated by the negative impact of a ‘distorted’ body image on depression. In this sense a ‘distorted’ body image might be remotely compared to physical disability which represents a potentially powerful confounding variable as it is associated with both a higher risk of depression and lower levels of physical activity [19]. A practical implication of this result could be that PA programmes targeting persons with depressive symptomatology should include a component to improve body perception.

A second interesting finding regarding body image as effect modifier is that low PA was positively associated with academic performance in students perceiving themselves as just right, but negatively in students perceiving themselves as overweight. This suggested that students performing well were more likely to be sedentary when they were satisfied with their weight/body image, but more likely to be physically active when they perceived themselves as overweight.

This study has limitations and generalization of the findings requires caution. As a cross-sectional survey, the findings are associations not causations, with difficulty in determining the direction of the effects. We surveyed seven universities in the UK, and despite the ‘broadening’ of our data collection in an attempt that the selection of students would be representative of their universities, our sample remains a convenience sample. Nevertheless, convenience samples are not uncommon in student surveys in Hong Kong [85], USA [86], or Australia [87]. Similarly, in the USA, universities and colleges self-selected themselves to participate in the American College Health Association National College Health Assessment survey [88]. Recall bias, sociability and social desirability could influence self-reported data. As the questionnaire was compiled from different published sources, we assessed some variables by single item measures and other variables by scales that had been validated by others. However, the necessity of a general student health survey to be conducted within a short time in classes, makes the use of in depth measures for each health factor unfeasible. Hence, we did not investigate other forms of PA that include work-related, transport-related and domestic-related activities. Nevertheless activities performed during leisure-time are generally more enjoyable and undertaken by personal choice.

## 5. Conclusions

An overall conclusion from this study is that the level of PA is relatively low in this sample of students from different British universities, when compared with students from the United States. This calls for effective measures and interventions at universities encouraging students to be more physically active, in maintaining and supporting an adequate level of PA. Such initiatives could include offering an attractive university sports programme, enabling active transport as well as launching PA campaigns and educational activities. We conclude from our study that PA is inversely associated with depressive symptoms. Such a positive effect of PA on mental health and well-being could well be addressed in these activities and programmes promoting PA. Individual counselling regarding PA for students with mental health problems should take potential body image distortion into account.

## Figures and Tables

**Table 1.** Characteristics of the sample: levels of PA and variables potentially associated with PA.

**Variable**	**N (valid %) of sample**
**Demographic and academic factors**	

Gender (male)	765 (22.1)
Age group (years)	
≤25 years	2534 (71.3)
>25 years	1020 (28.7)
Year at university	
1st year undergraduate	1491 (42.6)
2nd year undergraduate	1095 (31.3)
3rd year undergraduate	655 (18.7)

**Physical activity and muscle strengthening**	

Low PA^a^	764 (23.2)
Achieve recommended level of moderate PA^b^	420 (12.4)
Achieve recommended level of vigorous PA^b^	1131 (33.1)
Strengthening exercises (at least twice per week)^c^	743 (23.9)

**Depressive symptoms**	

M-BDI Depression score >4th quintile	1254 (39.4)
M-BDI Depression score >5th quintile	656 (20.6)

**Academic performance** relative to peers (better or much better)	547 (15.8)

**Self-rated health and health awareness**	

Perceived health (very good or excellent)	1706 (47.5)
Keeping an eye on one’s health (much or very much)	3007 (84.0)

**Body image**	

Body image perceived as underweight (a little or very)	265 (7.5)
Body image perceived as ‘just right’	1163 (32.9)
Body image perceived as ‘overweight’ (a little or very)	2110 (59.6)

a Low active = 0 vigorous and 0 or 1 day of moderate exercise in the past 7 days [81];

b as recommended by guidelines [75];

c [75].

**Table 2.** Multifactorial logistic regression models for factors associated with three different forms of PA as dependent variables.

	**Low PA**		**Moderate PA**		**Vigorous PA**	

**Variable**	OR^a^	95%CI	OR^a^	95%CI	OR^a^	95%CI
**Demographic factors**						

Gender (male)	0.85	0.64–1.14	**1.57**	**1.18–2.11**	**1.53**	**1.21–1.94**
Age	**1.02**	**1.01–1.04**	1.01	0.99–1.02	0.99	0.98–1.01

**Depressive symptoms**						

M–BDI score 1st quintile	1.00	0.74–1.43	1.00		1.00	
M–BDI score 2nd quintile	1.03	0.66–1.28	0.68	0.47–0.99	0.87	0.64–1.16
M–BDI score 3rd quintile	0.92	0.73–1.43	0.85	0.59–1.22	0.81	0.60–1.09
M–BDI score 4th quintile	1.02	0.95–1.84	0.90	0.62–1.32	**0.65**	**0.47–0.88**
M–BDI score 5th quintile	1.32	0.79–1.11	**0.64**	**0.42–0.99**	**0.59**	**0.42–0.82**

**Self-rated health and health awareness**						

Perceived health (from very poor to excellent)	**0.83**	**0.71–0.91**	**1.27**	**1.08–1.49**	**1.23**	**1.08–1.39**
Health awareness (from not all to very much)	**0.51**	**0.43–0.61**	**1.31**	**1.07–1.60**	**1.64**	**1.39–1.93**

**Muscle strengthening**						

Strengthening exercise (≥twice per week *vs.* less)	**0.15**	**0.10–0.23**	**3.02**	**2.33–3.92**	**8.25**	**6.60–10.33**

**Academic performance**						

Relative to peers (from much worse to much better)	0.94	0.79–1.11	0.99	0.82–1.22	0.96	0.82–1.13

a Odds ratios controlled for university, and all other variables in the table; values in bold indicate statistically significant findings.

**Table 3.** Regression models* for factors associated with three forms of PA as dependent variables by body image perception.

	**Students perceiving themselves as *just right***						**Students perceiving themselves as *overweight***					

	**Low PA**		**Moderate PA**		**Vigorous PA**		**Low PA**		**Moderate PA**		**Vigorous PA**	

**Variable**	OR^a^	95%CI	OR^a^	95%CI	OR^a^	95%CI	OR^a^	95%CI	OR^a^	95%CI	OR^a^	95%CI
**Demographic factors**												

Gender (male)	0.63	0.37–1.07	1.57	0.98–2.50	**1.49**	**1.01–2.19**	0.95	0.63–1.41	**1.79**	**1.16–2.75**	**1.65**	**1.15–2.36**
Age	1.01	0.99–1.04	1.01	0.98–1.03	1.01	0.98–1.03	**1.03**	**1.01–1.04**	1.01	0.98–1.03	0.99	0.97–1.01

**Depressive symptoms**												

M–BDI score 1st quintile	1.00	0.36–1.08	1.00		1.00		1.00		1.00		1.00	
M–BDI score 2nd quintile	0.62	0.41–1.30	0.41	0.24–0.72	0.85	0.55–1.33	1.22	0.78–1.91	0.86	0.48–1.53	0.75	0.48–1.18
M–BDI score 3rd quintile	0.73	0.62–2.06	0.68	0.39–1.19	0.95	0.59–1.53	0.91	0.59–1.40	0.99	0.58–1.70	0.81	0.53–1.23
M–BDI score 4th quintile	1.13	0.86–2.81	0.64	0.34–1.23	**0.36**	**0.20–0.63**	0.93	0.60–1.45	1.27	0.74–2.18	0.83	0.54–1.27
M–BDI score 5th quintile	1.55	0.86–2.81	**0.29**	**0.13–0.66**	**0.53**	**0.29–0.94**	1.15	0.75–1.77	0.92	0.51–1.65	**0.56**	**0.35–0.88**

**Self-rated health and health awareness**												

Perceived health (from very poor to excellent)	**0.71**	**0.55–0.90**	1.03	0.79–1.35	1.05	0.85–1.30	0.87	0.73–1.02	**1.31**	**1.04–1.64**	**1.29**	**1.09–1.54**
Health awareness (from not all to very much)	**0.49**	**0.35–0.67**	**1.76**	**1.26–2.45**	**1.52**	**1.17–1.97**	**0.54**	**0.43–0.68**	1.14	0.85–1.52	**1.86**	**1.47–2.35**

**Muscle strengthening**												

Strengthening exercise (at least twice per week vs less)	**0.15**	**0.07–0.30**	**3.35**	**2.19–5.13**	**8.98**	**6.11–13.21**	**0.14**	**0.08–0.25**	**3.27**	**2.26–4.73**	**9.48**	**6.91–12.99**

**Academic performance**												

Relative to peers (from much worse to much better)	**1.38**	**1.01–1.86**	0.82	0.59–1.16	0.88	0.68–1.14	**0.78**	**0.63–0.97**	1.24	0.93–1.65	0.97	0.78–1.21

* Multifactorial logistic regression;

a Odds ratios controlled for university, gender and age and all other variables in the table; values in bold indicate statistically significant findings.

## References

[1] Plante TG, Rodin J (1990). Physical fitness and enhanced psychological health. Curr. Psychol.

[2] Byrne A, Byrne DG (1993). The effect of exercise on depression, anxiety and other mood states: A review. J. Psychosom. Res.

[3] Dunn HL (1961). High Level Wellness.

[4] Paluska SA, Schwenk TL (2000). Physical activity and mental health. Sports Med.

[5] Brosse AL, Sheets ES, Lett HS, Blumenthal JA (2002). Exercise and the treatment of clinical depression in adults. Sports Med.

[6] Saxena S, Ommeren MV, Tang KC (2005). Armstrong TP: Mental health benefits of physical activity. J. Ment. Health.

[7] Craft LL, Perna FM (2004). The benefits of exercise for the clinically depressed. Prim. Care Companion J. Clin. Psychiatry.

[8] Teychenne M, Ball K, Salmon J (2008). Physical activity and likelihood of depression in adults: A review. Prev. Med.

[9] Galper DI, Trivedi MH, Barlow CE, Dunn AL, Kampert JB (2006). Inverse association between physical inactivity and mental health in men and women. Med. Sci. Sports Exerc.

[10] Brown WJ, Ford JH, Burton NW, Marshall AL, Dobson AJ (2005). Prospective study of physical activity and depressive symptoms in middle-aged women. Am. J. Prev. Med.

[11] Harris AHS, Cronkite R, Moos R (2006). Physical activity, exercise coping and depression in a 10-year cohort study of depressed patients. J. Affect. Disord.

[12] Dunn AL, Trivedi MH, Kampert JB, Clark CG, Chambliss HO (2005). Exercise treatment for depression: Efficacy and dose response. Am. J. Prev. Med.

[13] Dimeo F, Bauer M, Varahram I, Proest G, Halter U (2001). Benefits of aerobic exercise in patients with major depression: A pilot study. Br. J. Sports Med.

[14] Kull M (2002). The relationship between physical activity, health status and psychological well being of fertility-aged women. Scand. J. Med. Sci. Sports.

[15] Brown WJ, Mishra G, Lee C, Bauman A (2000). Leisure time physical activity in Australian women: Relationship with well being and symptoms. Res. Q. Exerc. Sport.

[16] Singh NA, Clements KM, Fiatarone MA (1997). A randomized controlled trial of progressive resistance training in depressed elders. J. Gerontol. A Biol. Sci. Med. Sci.

[17] Tolmunen T, Laukkanen JA, Hintikka J, Kurl S, Viinamaki H, Salonen R, Kauhanen J, Kaplan GA, Salonen JT (2006). Low maximal oxygen uptake is associated with elevated depressive symptoms in middle-aged men. Eur. J. Epidemiol.

[18] Hassmen P, Koivula N, Uutela A (2000). Physical exercise and psychological well-being: A population study in Finland. Prev. Med.

[19] Strawbridge WJ, Deleger S, Roberts RE, Kaplan GA (2002). Physical activity reduces the risk of subsequent depression for older adults. Am. J. Epidemiol.

[20] Farmer ME, Locke BZ, Moscicki EK, Dannenberg AL, Larson DB, Radloff LS (1988). Physical activity and depressive symptoms: The NHANES I epidemiologic follow-up study. Am. J. Epidemiol.

[21] Camacho TC, Roberts RE, Lazarus NB, Kaplan GA, Cohen RD (1991). Physical activity and depression: Evidence from the Alameda county study. Am. J. Epidemiol.

[22] Wiles NJ, Haase AM, Gallacher J, Lawlor DA, Lewis G (2007). Physical activity and common mental disorder: Results from the Caerphilly study. Am. J. Epidemiol.

[23] Cooper-Patrick L, Ford DE, Mead LA, Chang PP, Klag MJ (1997). Exercise and depression in midlife: A prospective study. Am. J. Public Health.

[24] Lennox SS, Bedell JR, Stone AA (1990). The effect of exercise on normal mood. J. Psychosom. Res.

[25] Weyerer S (1992). Physical inactivity and depression in the community: Evidence from the upper Bavarian field study. Int. J. Sports Med.

[26] Paffenbarger RS, Lee IM, Leung R (1994). Physical activity and personal characteristics associated with depression and suicide in American college men. Acta. Psychiatr. Scand. Suppl.

[27] Valtonen M, Laaksonen DE, Laukkanen J, Tolmunen T, Rauramaa R, Viinamäki H, Kauhanen J, Lakka T, Niskanen L (2009). Leisure-time physical activity, cardiorespiratory fitness and feelings of hopelessness in men. BMC Public Health.

[28] McCarty CA, Mason WA, Kosterman R, Hawkins JD, Lengua LJ, McCauley E (2008). Adolescent school failure predicts later depression among girls. J. Adolesc. Health.

[29] Zullig KJ, Valois RF, Drane JW (2005). Adolescent distinctions between quality of life and self-rated health in quality of life research. Health Qual Life Outcome.

[30] Sawatzky R, Ratner PA, Johnson JL, Kopec JA, Zumbo BD (2010). Self-reported physical and mental health status and quality of life in adolescents: A latent variable mediation model. Health Qual Life Outcome.

[31] Tanielian T, Jaycox LH, Paddock SM, Chandra A, Meredith LS, Burnam MA (2009). Improving treatment seeking among adolescents with depression: Understanding readiness for treatment. J. Adolesc. Health.

[32] Lysaker PH, Buck KD, Salvatore G, Popolo R, Dimaggio G (2009). Lack of awareness of illness in schizophrenia: Conceptualizations, correlates and treatment approaches. Expert. Rev. Neurother.

[33] Coleman L, Cox L, Roker D (2008). Girls and young women’s participation in physical activity: Psychological and social influences. Health Educ. Res.

[34] Oenema A, Brug J (2003). Feedback strategies to raise awareness of personal dietary intake: Results of a randomised controlled trial. Prev. Med.

[35] Eveland-Sayers BM, Farley RS, Fuller DK, Morgan DW, Caputo JL (2009). Physical fitness and academic achievement in elementary school children. J. Phys. Act. Health.

[36] Kwak LPJ, Kremers S, Bergman P, Ruiz JR, Rizzo NS, Sjöström M (2009). Associations between physical activity, fitness, and academic achievement. J. Pediatr.

[37] Chomitz VR, Slining MM, McGowan RJ, Mitchell SE, Dawson GF, Hacker KA (2009). Is there a relationship between physical fitness and academic achievement? Positive results from public school children in the northeastern United States. J. Sch. Health.

[38] Tomporowski PD, Davis CL, Miller PH, Naglieri JA (2008). Exercise and children’s intelligence, cognition and academic achievement. Educ. Psychol. Rev.

[39] Ross CE, Wu C (1995). The links between education and health. Am. Sociol. Rev.

[40] Mirowsky J, Ross CE (2003). Education, Social Status, and Health.

[41] Reinherz HZ, Giaconia RM, Hauf AMC, Wasserman MS, Silverman AB (1999). Major depression in the transition to adulthood: Risks and implications. J. Abnorm. Psychol.

[42] Needham BL (2009). Adolescent depressive symptomatology and young adult educational attainment: An examination of gender differences. J. Adolesc. Health.

[43] Cheung PCH, Ip PLS, Lam ST, Bibby H (2007). A study on body weight perception and weight control behaviours among adolescents in Hong Kong. Hong Kong Med. J.

[44] Schmitt M, Maes J (2000). Vorschlag zur Vereinfachung des Beck-Depressions-Inventars (BDI). Diagnostica.

[45] Schmitt M, Beckmann M, Dusi D, Maes J, Schiller A, Schonauer K (2003). Messgu‘te des vereinfachten Beck-Depressions-Inventars (BDI-V). Diagnostica.

[46] Beck AT, Steer RA, Ball R, Ranieri W (1996). Comparison of Beck Depression Inventories-IA and -II in psychiatric outpatients. J. Pers. Assess.

[47] El Ansari W, Stock C (2010). Is the health and wellbeing of university students associated with their academic performance? Cross sectional findings from the United Kingdom. Int. J. Environ. Res. Public Health.

[48] Teychenne M, Ball K, Salmon J (2008). Associations between physical activity and depressive symptoms in women. Int J Behav Nutr Phys Act.

[49] Balkin RS, Tietjen-Smith T, Caldwell C, Shen Y (2007). The utilization of exercise to decrease depressive symptoms in young adult women. Adultspan.

[50] Teychenne M, Ball K, Salmon J (2010). Physical activity, sedentary behavior and depression among disadvantaged women. Health Educ. Res.

[51] Hughes JR, Casal DC, Leon AS (1986). Psychological effects of exercise: A randomized cross-over trial. J. Psychosom. Res.

[52] King AC, Taylor CB, Haskell WL, Debusk RF (1989). Influence of regular aerobic exercise on psychological health: A radomized, controlled trial of healthy middle-aged adults. Health Psychol.

[53] Moses J, Steptoe A, Mathews A, Edwards S (1989). The effects of exercise training on mental well-being in the normal population: A controlled trial. J. Psychosom. Res.

[54] McLafferty C, Wetzstein C, Hunter G (2004). Resistance training is associated with improved mood in healthy older adults. Percept. Mot. Skills.

[55] (2008). Physical Activity Guidelines for Americans.

[56] Allgower A, Wardle J, Steptoe A (2001). Depressive symptoms, social support, and personal health behaviors in young men and women. Health. Psychol.

[57] Stephens T (1988). Physical activity and mental health in the United States and Canada: Evidence from four population surveys. Prev. Med.

[58] Wassertheil-Smoller S, Shumaker S, Ockene J, Talavera GA, Greenland P, Cochrane B, Robbins J, Aragaki A, Dunbar-Jacob J (2004). Depression and cardiovascular sequelae in postmenopausal women: The Women’s Health Initiative (WHI). Arch. Intern. Med.

[59] King AC, Taylor CB, Haskell WL (1993). Effects of differing intensities and formats of 12 months of exercise training on psychological outcomes in older adults. Health. Psychol.

[60] Doyne EJ, Ossip-Klein DJ, Bowman ED, Osborn KM, McDougall-Wilson IB, Neimayer RA (1987). Running *versus* weight lifting in the treatment of depression. J. Consult. Clin. Psychol.

[61] Lipowski M, Bulinski L, Krawczynski M (2009). Physical activities among other types of health-related behaviour in people losing weight. Med Sci Monit.

[62] Cunninghan MR, Roberts AR, Barbee AP, Druen PB (1995). “Their ideas of beauty are, on the whole, the same as ours”. Consistency and variability in the cross-cultural perception of female physical attractiveness. J. Pers. Soc. Psychol.

[63] Sparkes AC, Fox KR (1997). Reflections on the socially constructed physical self. The Physical Self: From Motivation to Well-Being.

[64] Fabricatore AN, Wadden TA, Sarwer DB, Faith MS (2005). Health-related quality of life and symptoms of depression in extremely obese persons seeking bariatric surgery. Obes. Surg.

[65] Kolotkin RL, Crosby RD, Williams GR (2002). Health-related quality of life varies among obese subgroups. Obes. Res.

[66] Pesa JA, Syre TR, Jones E (2000). Psychosocial differences associated with body weight among female adolescents: The importance of body image. J. Adolesc. Health.

[67] Buddeberg-Fischer B, Klaghofer R, Reed V (1999). Associations between body weight, psychiatric disorders and body image in female adolescents. Psychother. Psychosom.

[68] Swallen KC, Reither EN, Haas SA, Meier AM (2005). Overweight, obesity, and health-related quality of life among adolescents: The National Longitudinal Study of Adolescent Health. Pediatrics.

[69] Carpenter KM, Hasin DS, Allisonm DBL, Faith MS (2000). Relationship between obesity and DSM-IV major depressive disorder, suicide ideation, and suicide attempts: Results from a general population study. Am. J. Public Health.

[70] Roberts RE, Kaplan GA, Shema SJ, Strawbridge WJ (2000). Are the obese at greater risk for depression?. Am. J. Epidemiol.

[71] Roberts RE, Strawbridge WJ, Deleger S, Kaplan GA (2002). Are the fat more jolly?. Ann. Behav. Med.

[72] Caspersen CJ, Powell KE, Christenson GM (1985). Physical activity, exercise, and physical fitness: Definitions and distinctions for health-related research. Public Health Rep.

[73] El Ansari W, Maxwell AE, Mikolajczyk RT, Stock C, Naydenova V, Kraemer A (2007). Promoting public health: Benefits and challenges of a European wide research consortium on student health. Cent. Eur. J. Public Health.

[74] El Ansari W, Clausen SV, Mabhala A, Stock C (2010). How do I look? Body image perceptions among university students from England and Denmark. Int. J. Environ. Res. Public Health.

[75] Haskell WL, Lee IM, Pate RR, Powell KE, Blair SN, Franklin BA, Macera CA, Heath GW, Thompson PD, Bauman A (2007). Physical activity and public health: Updated recommendation for adults from the American College of Sports Medicine and the American Heart Association. Circulation.

[76] Schmitt M, Altstoter-Gleich C, Hinz A, Maes J, Brahler E (2006). Normwerte fur das Vereinfachte Beck-Depressions-Inventar (BDI-V) in der Allgemeinbevolkerung. Diagnostica.

[77] Currie C, Sandal O, Boyce W, Smith R (2001). Health Behaviour in School-aged Children: A WHO Cross-National Study (HBSC), Research Protocol for the Survey 2001/2002.

[78] Potthoff P, Schroeder E, Reis U, Klamert A (1999). Process and results of field work concerning the Federal Health Survey. Gesundheitswesen.

[79] American College Health Association (2006). National College Health Assessment (ACHA-NCHA) Spring 2005 Reference Group Data Report (Abridged). J Am Coll Health.

[80] Stock C, Kucuk N, Miseviciene I, Guillen-Grima F, Petkeviciene J, Aguinaga-Ontoso I, Krämer A (2003). Differences in health complaints between university students from three European countries. Prev. Med.

[81] Seo DC, Nehl E, Agley J, Ma SM (2007). Relations between physical activity and behavioral and perceptual correlates among Midwestern college students. J. Am. Coll. Health.

[82] Lowry R, Galuska DA, Fulton JE, Wechsler H, Kann L, Collins JL (2000). Physical activity, food choice, and weight management goals and practices among US college students. Am. J. Prev. Med.

[83] American College Health Association (2007). American College Health Association National College Health Assessment Spring 2006 Reference Group Data Report (Abridged). J. Am. Coll. Health.

[84] Mikolajczyk RT, Maxwell AE, El Ansari W, Naydenova V, Stock C, Ilieva S, Dudziak U, Naydova I (2008). Prevalence of depressive symptoms in university students from Germany, Denmark, Poland and Bulgaria. Soc. Psychiatry Psychiatr. Epidemiol.

[85] Lee RL, Loke AJ (2005). Health-promoting behaviors and psychosocial well-being of university students in Hong Kong. Public Health Nurs.

[86] Richards A, Kattelmann KK, Ren C (2006). Motivating 18- to 24-year-olds to increase their fruit and vegetable consumption. J. Am. Diet Assoc.

[87] Hsieh PL (2004). Factors influencing student‘s decisions to choose healthy or unhealthy snacks at the University of Newcastle, Australia. J. Nurs. Res.

[88] American College Health Association (2006). American College Health Association National College Health Assessment (ACHA-NCHA): Spring 2005 Reference Group Report (Abridged). J. Am. Coll. Health.

